# Diving behaviour of Cuvier's beaked whales exposed to two types of military sonar

**DOI:** 10.1098/rsos.170629

**Published:** 2017-08-30

**Authors:** Erin A. Falcone, Gregory S. Schorr, Stephanie L. Watwood, Stacy L. DeRuiter, Alexandre N. Zerbini, Russel D. Andrews, Ronald P. Morrissey, David J. Moretti

**Affiliations:** 1 Marine Ecology and Telemetry Research, 2420 Nellita Rd NW, Seabeck, WA 98380, USA; 2 Naval Undersea Warfare Center Division, Code 74, Newport, RI 02840, USA; 3 Department of Mathematics and Statistics, Calvin College, 1740 Knollcrest Circle SE, Grand Rapids, MI 49546, USA; 4 Marine Mammal Laboratory, Alaska Fisheries Science Center, NMFS-NOAA, 7600 Sand Point Way NE, Seattle, WA 98115, USA; 5 Cascadia Research Collective, 218 ½ W 4th Avenue, Olympia, WA 98501, USA; 6 College of Fisheries and Ocean Sciences, University of Alaska Fairbanks, PO Box 757220, Fairbanks, AK 99775, USA

**Keywords:** Cuvier's beaked whale, tagging, sonar, California, behavioural response

## Abstract

Cuvier's beaked whales (*Ziphius cavirostris*) have stranded in association with mid-frequency active sonar (MFAS) use, and though the causative mechanism linking these events remains unclear, it is believed to be behaviourally mediated. To determine whether MFAS use was associated with behavioural changes in this species, satellite tags were used to record the diving and movements of 16 Cuvier's beaked whales for up to 88 days in a region of frequent MFAS training off the coast of Southern California. Tag data were combined with summarized records of concurrent bouts of high-power, surface-ship and mid-power, helicopter-deployed MFAS use, along with other potential covariates, in generalized additive mixed-effects models. Deep dives, shallow dives and surface intervals tended to become longer during MFAS use, with some variation associated with the total amount of overlapping MFAS during the behaviour. These changes in dives and surface intervals contributed to a longer interval between deep dives, a proxy for foraging disruption in this species. Most responses intensified with proximity and were more pronounced during mid-power than high-power MFAS use at comparable distances within approximately 50 km, despite the significantly lower source level of mid-power MFAS. However, distance-mediated responses to high-power MFAS, and increased deep dive intervals during mid-power MFAS, were evident up to approximately 100 km away.

## 1. Introduction

Beaked whale species (Family Ziphiidae) have been observed to strand in association with the use of mid-frequency active sonars (hereafter ‘MFAS’), such as those used by military vessels around the world to search for quiet submarines [1–3]. Stranded whales have displayed gas bubble-associated lesions and fat emboli similar to decompression sickness (DCS) symptoms in human divers, to which marine mammals were previously thought immune [4–6]. Despite mounting public concern and considerable research in the years since these stranding events were first observed [7–12], the mechanism through which MFAS exposure has led to DCS-like syndrome in these whales remains unclear [13], as do any sublethal individual effects and population-level impacts that may arise from long-term, cumulative exposures. Consequently, current methods for estimating the impacts of MFAS use on beaked whales involve considerable uncertainty, and few suitable approaches have been identified to mitigate these risks.

One inherent challenge in studying the relationship between beaked whales and MFAS is the nature of the whales themselves. Beaked whales were minimally studied prior to 1999, when interest in these species increased following the aforementioned MFAS-associated stranding events. Tagging studies revealed that these deep-water cetaceans are prodigious divers, and that they tend to perform a stereotypic pattern of deep, foraging dives separated by a series of shallower, non-foraging dives [14,15]. Of the small number of species that have been fitted with dive-recording tags, Cuvier's beaked whales (*Ziphius cavirostris*), in particular, have conducted dives far exceeding those of any other mammal in both depth and duration [16].

Cuvier's beaked whales are also the species that has most frequently stranded in association with MFAS use [3]. This coincidence of extreme, yet stereotypic, diving behaviour and apparent sensitivity to MFAS has suggested that these mortalities may be behaviourally mediated [13]. That beaked whales alter their behaviour in response to simulated and real MFAS use, at both individual and group levels, is no longer generally questioned. At the Atlantic Undersea Test and Evaluation Center (AUTEC) in the Bahamas, for example, shifts in Blainville's beaked whale (*Mesoplodon densirostris*) vocal activity suggest that whales move off the testing range during multi-day, multi-ship exercises that include MFAS use [9,10,17]. Two Cuvier's beaked whales tagged as part of a controlled-exposure experiment near the Southern California Anti-submarine Warfare Range (SOAR) rapidly moved away from simulated MFAS at close range (3.4–9.5 km) and low received sound levels, and ceased or did not initiate foraging in the hours following exposure [11]. However, one of these tagged whales did not respond to distant (greater than 40 km) high-power MFAS from a naval ship at comparable received levels to the simulated signal, suggesting that source proximity, in addition to received level, influences response [11]. These behavioural changes (foraging disruption and displacement from foraging habitats) have potential long-term consequences if repeated exposures result in a reduction in individual energy stores [17,18]. Individual beaked whales have exhibited long-term site fidelity to both AUTEC and SOAR despite regular MFAS use there [19,20], suggesting they do not routinely respond to MFAS on these ranges in a manner that leads directly to mortality. Therefore, it is important to understand how and when they do respond, and the long-term, cumulative effect that these repeated responses may have on individuals and populations.

**Figure 1.**
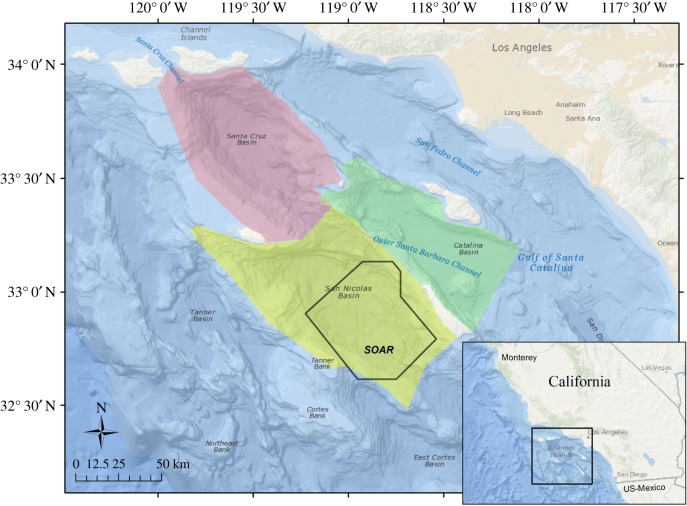
Map of study area with SOAR outlined in black and the geographical sub-areas used in analysis, corresponding to the three primary basins used by tagged whales, indicated by colour. Locations not within these basin boundaries were assigned to ‘Outside’. Inset map in the lower right corner shows a larger geographical area around the study site.

Understanding the effects of repeated exposure is particularly relevant off the coast of southern California, given that photographed and satellite-tagged Cuvier's beaked whales have demonstrated prolonged and repeated use of offshore basins there [16,19]. MFAS is used regularly throughout the broad Southern California Range Complex (SOCAL), though exercises that include it are often concentrated on or near SOAR (figure 1); this is an approximately 2200 km^2^ area that extends west from San Clemente Island throughout much of the San Nicolas Basin and contains 117 bottom-mounted hydrophones. In addition to the high-power, hull-mounted MFAS that has received the most research attention to date, these exercises also often include less powerful sound sources, such as helicopter-deployed, dipping MFAS systems, active sonobuoys, acoustic countermeasures and pingers [21]. Training occurs year-round in SOCAL and on SOAR, in configurations ranging from single platform exercises lasting less than an hour to multi-day coordinated events including multiple ships, helicopters and other sound sources. There are also exercises that do not include sonar, but which do include other noise-generating activities such as explosions and arms fire [21].

Satellite-tagged Cuvier's beaked whales in southern California can provide sizeable samples of diving behaviour during time periods with and without MFAS use, across the broad range of spatial and temporal contexts at which exposures and responses may occur. Here, we combined extended records of movements and diving patterns from Cuvier's beaked whales satellite-tagged on or near SOAR from 2011 to 2015 with coincident records of MFAS use in the region, including both the high-power surface-ship MFAS that has been the focus of earlier behavioural response research and mid-power dipping MFAS, the effects of which have received little prior study. We also identified periods that were confirmed free of MFAS use. The resulting dataset afforded the first opportunity to characterize the diving behaviour of whales in this region in the presence and absence of two commonly used military MFAS systems, including effects of source–whale distance and amount of overlapping sonar use during dives.

## 2. Material and methods

### 2.1. Behavioural data collection

Cuvier's beaked whales were tagged with Wildlife Computers SPLASH10 transmitters in the Low Impact Minimally Percutaneous External-electronics Transmitter (LIMPET) configuration, which were programmed and deployed as described by Schorr *et al.* [16]. Location estimates of tagged whales were provided by the Argos system based on the least-squares estimation method. Tags transmitted a record of diving behaviour in the form of a ‘Behaviour Log’ (BL). Each BL record represented a summarized ‘Dive’ or ‘Surfacing’ event: dives were recorded when the whale exceeded the user-defined threshold of greater than 50 m depth for greater than 30 s duration and each dive record included the start and end time, the maximum depth reached, the total duration and an approximate dive shape based upon the proportion of time spent below 80% of the maximum recorded depth [22] (see electronic supplementary material, figure S1). Surfacing events summarized the period of time between each qualifying dive. All dives were passed through a K-means cluster analysis, which assigned them into either ‘deep’ or ‘shallow’ classes as a function of maximum depth and total duration, on a per-individual basis, to control for individual behavioural variation.

BL data were recovered via the Argos satellite system and, beginning in 2014, a land-based Argos receiving station (the Wildlife Computers Mote) installed on San Clemente Island; this significantly improved BL data throughput in later tags. Argos location estimates were filtered using the Douglas Argos Filter [23] with settings defined in [16]. All filtered locations were fitted by a continuous-time correlated random walk movement model using the R package *crawl* [24] to estimate each whale's position every 30 min with a 95% CI around each location estimate. The resultant modelled locations were passed through the Mysticetus Observation System (v.#1.8.0.124, Entiat River Technologies, Preston, WA, USA) to extract the bottom depth, slope and aspect, to assign locations to user-defined geographical sub-areas (figure 1) and to determine whether each modelled location fell within the SOAR boundaries. Each dive and surfacing event in the BL was associated with the modelled location that occurred closest in time to the event start. The start time and location of each BL event were passed through a solar calculator (http://www.esrl.noaa.gov/gmd/grad/solcalc/calcdetails.html) to estimate the solar elevation at the event start; solar elevation data were used to classify each event as starting in the Dawn (solar elevation from −12 to +6 degrees of the horizon, bracketing sunrise), Dusk (+6 to −12 degrees, bracketing sunset), Day (greater than +6 degrees) or Night (less than −12 degrees) periods.

### 2.2. Sonar data collection and compilation

Two sources of MFAS use data from Southern California were referenced for this study. The first was the SOAR hydrophone array. Marine mammal passive acoustic detection reports from SOAR hydrophones have been archived since 2008 as part of the Marine Mammal Monitoring on Ranges (M3R) programme [25]. In addition to detection reports of vocalizing animals, M3R archives can provide an accurate record of MFAS used on or near SOAR, and just as importantly, a definitive record of times when no high- or mid-power sonar is present. The M3R data processing procedures are described fully in Jarvis *et al.* [25]. The analogue output from each hydrophone is digitized. A Fast Fourier Transform (FFT) with a rectangular window and 50% overlap (a sampling frequency of 96 kHz results in a frequency resolution of 46.875 Hz and a time step of 10.67 ms) is performed on each hydrophone channel. An adaptive, noise-variable threshold based on an exponential average is run on every bin of the FFT. Bins with energy above the threshold are set to 1 and those below to 0. A detection report is generated for each FFT from each hydrophone. Each report contains the FFT start time, hydrophone number, hard-limited binary frequency bin data (0/1), sampling frequency, FFT size, peak frequency and number of bins above the threshold. These detection reports are incorporated into the M3R archives. To verify transient signals in both real-time and post-recording, the ‘hard-limited’ FFT data are combined to provide a two-dimensional spectrogram display for a given hydrophone on demand.

The FFT detector reports were processed through an automated sonar detector that outputs the occurrence of MFAS signals, or pings, within a specified frequency band and time duration. The start time, number of FFT bins, duration, peak frequency and peak level for signals that exceeded the frequency and duration (number of consecutive FFT bins) thresholds were provided. The MFAS detector created a record for any signal with a duration longer than 1 s within the MFAS frequency bands; the actual signals transmitted during MFAS operations are not fixed, but are selected based on daily conditions and the objective of the operation (see electronic supplementary material, figure S2). Therefore, false positive detections (particularly of delphinid whistles) were common. Additionally, the detector reports all detections on any hydrophone; it does not correlate detections of the same signal across multiple hydrophones. To filter out false detections, the MFAS detector output was plotted in Matlab 2015a (Mathworks, Inc., Natick, MA) in two ways: (i) detection time versus peak level and (ii) detection time versus time since the last detection on the same hydrophone (inter-detection interval). Given the array geometry, if the source was on SOAR, it was typically within 3 km of the nearest hydrophone. Therefore, distinct, high-level pings were detected at regular intervals (usually every 20–30 s) consistent with typical MFAS transmissions. Detection of pings from sources outside SOAR could result in lower received levels, but the inter-detection interval and source frequencies also remained consistent with MFAS transmissions.

For each MFAS period detected through visual inspection of the output plots, the start time, end time, closest hydrophone (based on ping arrival times) and an estimate of type (as designated in the SPORTS database; see below) were recorded. An MFAS period was considered continuous provided the inter-detection interval on any single hydrophone was less than 3 min. For platforms that moved during transmission, the closest hydrophone was updated throughout the MFAS period. Periods with multiple overlapping MFAS sources were distinguished by (i) ‘spread’ in the inter-detection interval (due to arrival time differences between the two sources on different hydrophones) and (ii) rapidly changing closest hydrophone.

M3R archives were manually reviewed using two-dimensional spectrograms for most periods of detections to confirm the presence, location and type of MFAS. Archives were always manually reviewed for times when the detector output plots indicated multiple overlapping sources. MFAS type was determined by signal characteristics and by localization to a known platform reported in the area in the Range Operations Center event schedule (helicopter, guided missile destroyer, guided missile frigate, etc.). Custom M3R localization software was used to determine the location of MFAS detections by calculating time of arrival differences on a minimum of three manually selected non-collinear hydrophones [26]. Detection localizations were then compared to the recorded track positions of event vessels provided by the Range Operations Center, when available, to confirm source platform type.

All positive sonar detections from 2012 and 2014, and portions of detections from 2011 and 2015 where the output indicated multiple sources were present were manually reviewed. No false positive detections were identified after the earlier filtering steps described above. To estimate the false negative rate, periods with no sonar detections in the automated detector output were randomly selected and manually reviewed. Any periods where sonar was visually seen on a spectrogram of the hard-limited binary frequency bin data were noted and corrected in the data.

The second source of sonar use data was the US Navy's internal Sonar Positional Reporting System (SPORTS) database. SPORTS contains reports of sonar use from throughout the broad SOCAL operating area that are summarized into periods during which a sonar signal is repeatedly broadcast from a single platform at regular intervals (hereafter ‘bouts’). Each SPORTS report included the start time, start latitude and longitude, end time and sonar source type. ‘High-power’ source types in SPORTS include hull-mounted surface-ship MFAS, such as the AN/SQS-53C, with a published nominal frequency of 3.5 kHz and source level of 235 dB re 1 µPa @ 1 m (dB) [27]. ‘Mid-power’ source types refer to dipping MFAS, such as the AN/AQS-22, which is similar to surface-ship MFAS, but with a published nominal frequency of 4.1 kHz and source level of 217 dB [27]. The SPORTS database was queried for bouts of high-power and mid-power MFAS use during periods when tags were transmitting. All SPORTS reports with a start position outside the boundaries of SOAR were classified as such. Because SPORTS records are both manually captured at sea and later manually entered into the database, there are several steps where transcription errors can occur. Suspect reports (e.g. unusual locations, durations or movements when subsequent reports were known to come from the same platform) were provided to SPORTS data managers and corrected at their direction when possible. In the final SPORTS dataset, 22% of high-power MFAS-use records and 15% of mid-power MFAS-use records exceeded the maximum durations recorded in the archives for the same sonar type, including 15 records that reported sonar use for 24 h or more. While some of these records may have over-reported actual usage, under-reporting MFAS use was presumably also possible, but not readily detectable. Ultimately SPORTS represented the best available record of MFAS use outside SOAR, even though inaccurate reporting could reduce the apparent effect of exposure in this analysis (e.g. unexposed behaviours could be falsely associated with MFAS use and exposed behaviours could be included in MFAS-free data).

Because high-resolution, ping-level MFAS data from the archive were combined with low-resolution, summarized MFAS data from SPORTS to recreate a comprehensive record of use, continuous periods of MFAS use originating from the same platform in the archive were summarized into bouts as reported in SPORTS (start time and location, end time, type). When an accurate localization could not be derived for detections originating outside SOAR, they were associated with the position of the nearest edge phone on which the signal was detected as the best available location. Though the positions associated with these off-SOAR detections were known to be inaccurate, they provided a general location for screening against concurrent bouts in SPORTS. The two datasets were then compared to identify probable duplicate records (i.e. bouts that were reported in SPORTS and also recorded in the M3R archive) by calculating the differences in start time, end time, total duration of use and distance between the starting positions for all bouts of MFAS of the same type in each data source. Any bouts of the same sonar type that differed by less than 15 min in any time metric (start time, end time or duration of use) and started within 10 km of each other were identified as potential duplicates; wide intervals were used to capture any cases where variable detection in the archive or reporting in SPORTS (e.g. ‘lumping’ versus ‘splitting’ sequential bouts in rapid succession in one data source or the other) may have made the same sonar use appear different between the two sources. The relative timing and positions of the overlapping bouts, along with those immediately preceding and following them, were manually reviewed. Where the total available data suggested likely duplication between the data sources, a single record of the bout was retained from the archive if on SOAR and from SPORTS if not. The final combined dataset used in analysis included: all on-SOAR MFAS use detected in the archive; all off-SOAR MFAS use reported to SPORTS; any off-SOAR MFAS use detected in the archive that was not reported in SPORTS (with low-accuracy positions); and any on-SOAR MFAS use that was reported in SPORTS during periods when the archive was unavailable. The dataset excluded any on-SOAR SPORTS reports that occurred during MFAS-free periods confirmed by the archive. It also explicitly included all spans of time when neither high- nor mid-power MFAS was detected on SOAR, though records of concurrent off-SOAR use reported in SPORTS during these periods were retained.

### 2.3. Behavioural and sonar data integration

The combined, reconciled MFAS data were then integrated with the BL data from the tags. Each BL record was screened for temporal overlap with bouts of MFAS use, and, where found, the distance between the estimated position of the tagged animal and the start position of each overlapping MFAS bout was calculated (Dist), as was the proportion of the behavioural event (dive or surfacing) during which that MFAS source was in use (overlap ‘OL’, ranging from 0 if no MFAS was in use to 1 if MFAS was in use during the entire behaviour). Additionally, a vector was created between each whale position and the start position of any overlapping MFAS bouts, and the shallowest depth along the vector was extracted from an etopo2v2 bathymetry grid using ArcGIS v. 10.3.1. Any vectors that crossed emergent land masses (i.e. shallowest depth along vector greater than 0) were used to exclude that exposure under the premise that any sound emitted would probably have been blocked or severely attenuated at the whale. For modelling purposes, when multiple bouts of the same sonar type occurred during a single behavioural event, whether from a single platform transmitting sequentially or separate platforms, use was summarized for each sonar type as presence/absence, distance to the nearest transmission and combined overlap with all platforms (i.e. the sum of all overlapping minutes of sonar use/behaviour duration).

The following behavioural parameters were assessed for variation as a function of time of day, geographical area and MFAS exposure: deep dive duration, shallow dive duration, shallow dive depth, surface interval duration and the inter-deep-dive interval (IDDI, the duration from the end of one deep dive to the start of the next). MFAS exposure for the IDDI was summarized from the start of the deep dive that preceded the interval to the start of the deep dive that ended the interval in order to account for exposure at any time in the deep dive cycle. Two sets of data were created for each behavioural parameter: (i) data where both the whale and the transmitting platform were within the boundaries of SOAR (the ‘SOAR’ dataset) and (ii) the ‘Complete’ dataset, which included all behavioural and MFAS data regardless of the location of the whale or the platform. The SOAR dataset, selected for temporal and spatial accuracy, included only the highest confidence MFAS use data. This dataset included only a relatively small number of unconfirmed MFAS bouts that were reported in SPORTS when the archive was unavailable (17 high-power and 39 mid-power), and all MFAS-free periods were confirmed as such. Behavioural events during periods when MFAS use was not reported in SPORTS, but for which archives were not available to verify that SOAR was truly MFAS-free, were omitted from the SOAR dataset due to uncertainty.

The Complete dataset, which was selected for sample size, but subject to greater uncertainty in MFAS use, increased the range of whale–MFAS distances in the analysis by adding both behaviour and MFAS data from outside SOAR to the SOAR dataset. It included all known exposures except those that occurred with a land mass between the whale and the transmitting platform. Also included were behavioural events from periods when MFAS was not reported in SPORTS, but for which its absence could not be verified because the whale was either outside SOAR or inside range boundaries during a period when the archives were not recorded. The Complete dataset included geographical area as a covariate by assigning each behaviour to a sub-area within the region (San Nicolas Basin, Catalina Basin, Santa Cruz Basin or Outside these basins) (figure 1).

### 2.4. Data analysis

Each behavioural dataset was analysed using generalized additive mixed-effects models (GAMMs) fitted in R statistical computing software [28] using the gamm4 (gamm4 function; [29]) and MuMIn (uGamm and dredge functions; [30]) packages. Models were fitted for each of the five response variables of interest (deep dive duration, shallow dive duration, shallow dive depth, surfacing interval duration and IDDI), with separate models fitted to the SOAR and Complete datasets in each case [31]. IDDI observations were weighted (by IDDI divided by minimum IDDI) to account for a bias towards shorter intervals created by gaps in the BL. Intervals that included gaps (i.e. where one or more Argos messages were not recovered, and thus behaviour during the period was not documented) were excluded from the IDDI dataset; long intervals were more likely to include gaps, and thus be excluded. Each model included two candidate parametrizations for the effects of MFAS on the response variables. First, we fitted models in which the effect of each MFAS type (high- and/or mid-power) was a smooth function of source-to-whale distance (specifically, each smooth term was a shrinkage cubic regression spline with a basis of dimension 5, specified in R using the function ‘s’ from the mgcv package with bs = ‘cs’ and k = 5). For cases with no MFAS, this smooth term was multiplied by 0 using the ‘by’ input to the ‘s’ function, with the source–whale distance set to an arbitrarily large value of 500 km to avoid numerical problems. We also fitted models in which the presence of MFAS had constant effects (regardless of distance) by including a categorical predictor variable with values: no MFAS, high-power present, mid-power present or both types present. We compared models where both high- and mid-power MFAS had distance-dependent effects to models where both had constant effects. In addition, for all response variables except surface intervals, which were so short relative to most MFAS bouts that most OL values were 1, we included OL as another potential MFAS-related linear numerical predictor in each model. As appropriate for each response variable and dataset, we also considered time of day, region and surface sequence (categorical position of the surface interval in the deep dive cycle, e.g. immediately following a deep dive, between shallow dives, immediately preceding a deep dive, etc.) [16] as potential categorical predictors; and dive depth or dive duration as potential (linear) numerical predictors (table 4). To account for differences between whales, all models also included a random effect of individual whale. In cases where inspection of a plot of the residual autocorrelation function indicated that this random effect was not sufficient to reduce temporal residual autocorrelation to acceptable levels, we achieved the reduction by adding a second (nested) random effect of shorter time periods (2, 3, 4, 6 or 12 h) within whales, selecting the single time period that minimized Akaike's information criterion (AIC) for the full model. For each response variable, within each of the Complete and SOAR datasets, AIC was minimized to select the best model, considering all possible combinations of candidate predictors. AIC weight was used to indicate the relative probability of the best model over competing models for each response variable and dataset [32]. Prediction plots were generated to represent the shape and magnitude of the effect for each MFAS predictor included in each best model. For these plots, the values of all other predictors in each model were fixed at the median (for numerical predictors) or modal value (categorical predictors) from no sonar periods. In all prediction plots, the time of day was fixed at ‘Night’, and where Basin was included in a best model of the Complete dataset, its value was fixed at ‘San Nic’. Other fixed values varied among models and are included in each figure caption.

## 3. Results

Behavioural data from the 16 tagged whales included in the study are summarized in table 1. The reconciled MFAS data contained 790 bouts of high- and mid-power use totalling 913 h, which are summarized by data source, type and geographical area in table 2. It also included 369 MFAS-free periods on SOAR, totalling 4867 h. A subset of 122 of these periods identified by the automated sonar detector (983.4 h total, with 257 h from 2011, 492 h from 2012, 118 h from 2014 and 115 h from 2015) were manually reviewed for false negative detections. In 2011, MFAS of either type was detected on SOAR in manual review during 5.45% of the time that the detector classified as MFAS-free. In subsequent years, the false negative rate was reduced to 0.46%. Most false negatives resulted from one or two pings at the beginning of a successfully detected bout that were missed (and thus the length of the bout increased slightly), or cases where the sonar had used parameters different from those that were used to set the detector. Additionally, the automatic gain control settings on the hydrophones in operation prior to 2012 occasionally resulted in received levels which did not meet the parameters set in the sonar detector.

**Table 1.** Tag deployment summary by whale, with individual sample sizes for each behavioural response variable. The percentage of behaviours in each sample that coincided with MFAS use is provided in parentheses.

tagID	deployment date	days SOAR/total days	BL data (hrs)	deep dives (%MFAS)	shallow dives (%MFAS)	surface intervals (%MFAS)	inter-deep-dive intervals (%MFAS)
14	6 Jan 2011	13/23	91.2	32 (13%)	143 (4%)	175 (3%)	11 (18%)
15	6 Jan 2011	17/71	1015.9	315 (20%)	1609 (17%)	1923 (15%)	249 (31%)
16	6 Jan 2011	68/89	857.5	263 (18%)	1053 (14%)	1317 (11%)	176 (27%)
19	15 Jan 2012	12/12	172.5	53 (9%)	278 (13%)	330 (12%)	35 (14%)
20	15 Jan 2012	26/26	441.2	138 (16%)	646 (11%)	784 (11%)	105 (30%)
21	29 Mar 2013	17/47	625.6	199 (8%)	1070 (7%)	1271 (6%)	138 (12%)
22	30 Mar 2013	28/28	256.5	92 (9%)	445 (5%)	535 (4%)	42 (10%)
23	30 Mar 2013	7/7	97.7	31 (0%)	139 (1%)	171 (1%)	25 (4%)
24	4 Jan 2014	8/12	161.0	54 (2%)	217 (1%)	271 (1%)	37 (0%)
25	4 Jan 2014	8/8	90.7	28 (0%)	127 (0%)	153 (0%)	20 (0%)
26	7 Jan 2014	0/47	820.9	280 (6%)	1373 (5%)	1651 (4%)	242 (20%)
28	11 Jan 2014	49/49	1091.0	380 (9%)	1672 (8%)	2050 (6%)	363 (23%)
34	3 Jan 2015	16/16	385.7	122 (2%)	572 (3%)	693 (1%)	121 (6%)
35	7 Jan 2015	10/14	87.2	33 (3%)	124 (3%)	158 (2%)	22 (5%)
36	9 Jan 2015	19/43	932.6	289 (8%)	1371 (8%)	1658 (5%)	272 (16%)
37	9 Jan 2015	14/14	322.2	93 (3%)	478 (6%)	570 (2%)	92 (12%)

**Table 2.** Summary of MFAS use data, including the number of bouts and the bout duration (minutes) by data source, MFAS type and location.

		no. bouts		median bout duration (range)	
source	type	*Outside*	*SOAR*	*Outside*	*SOAR*
archive	high-power	28	66	32 (1–187)	67 (2–449)
	mid-power	32	289	4 (1–52)	7 (1–81)
SPORTS	high-power	251	20	79 (1–1439)	171 (0–1439)
	mid-power	57	47	7 (3–1439)	5 (0–64)

The combined behaviour–MFAS database contained 2189 dives and surface intervals (8.2% of all recorded) that occurred while high- or mid-power MFAS was being used at distances in the range of 1.9–419.4 km to the whale. The data included 1950 IDDIs, 303 (15.5%) of which coincided with, or immediately followed a deep dive that included high- or mid-power MFAS use. The distribution of exposures by response variable is summarized in table 3. Of 1943 dives and surface intervals that were exposed to high-power sonar, 312 (16%) coincided with more than one bout of sonar use. Overlap with multiple bouts of high-power MFAS occurred at similar rates in the sample of exposed deep and shallow dives (both approx. 17%) and surface intervals (15%), because high-power bouts were typically longer than all three behaviours [16] (table 2), and exposure to multiple bouts of high-power MFAS was most often due to multiple ships transmitting simultaneously. By contrast, overlap with multiple bouts of mid-power MFAS in any exposed behaviour was more common, though the frequency varied considerably between exposed deep dives (57%), shallow dives (38%) and surface intervals (5%). This was because mid-power MFAS was often used in a series of short bouts (table 2) that were shorter than most deep dives and many shallow dives, but longer than most surface intervals. Overlap with multiple bouts of either MFAS type was very common in the sample of exposed IDDIs due to the typically long duration of these intervals, with 131 (54% of total exposed to high-power) including more than one bout of high-power, and 76 (84% of total exposed to mid-power) more than one bout of mid-power MFAS use.

**Table 3.** Summary of sonar exposures by response variable and dataset.

			MFAS exposure			
response variable	dataset	total	high	mid	both	none^a^
deep dive duration	Complete	2402	202	33	12	2155
	SOAR	1137	13	15	0	1109
shallow dive duration	Complete	11 317	766	157	56	10 338
	SOAR	5132	59	91	8	4974
shallow dive depth	Complete	11 317	766	157	56	10 338
	SOAR	5132	59	91	8	4974
surface interval	Complete	13 710	825	104	33	12 748
	SOAR	6228	60	49	4	6115
inter-deep-dive interval	Complete	1950	213	65	25	1647
	SOAR	888	11	43	3	831

^a^‘None’ indicates behaviours with no reported MFAS use in the Complete dataset, and behaviours that were confirmed free of high- and mid-power MFAS use in the SOAR datasets.

Non-MFAS predictors were included in the best models for all behaviours (table 4). These included the dive depth when dive duration was the response variable, and dive duration when dive depth was the response variable. Position in the deep dive cycle was an important predictor of surface interval duration (surface intervals immediately preceding deep dives were longer than other types), and the IDDI duration was predicted by the duration of the preceding deep dive (longer dives tended to be followed by longer intervals). Time of day was included in the best models of all behaviours tested. Regional variability (by basin) was apparent in the Complete datasets for all behaviours except surface interval. There were no best models that included non-MFAS predictors for one dataset (Complete or SOAR), but not the other. In addition to these factors, at least one MFAS predictor was included in the best model for each response variable. Model selection results are summarized for each response variable and dataset in table 4, and described below.

**Table 4.** Predictors included in the best models by response variable and dataset. ‘High’ represents high-power MFAS sources, and ‘Mid’ mid-power sources. ‘Dist’ is the distance from the whale to the nearest source of that type, ‘Pres’ is the presence/absence of the source type at any distance, and ‘OL’ is the proportion of the behaviour that overlapped with MFAS use of the type. AIC weight of the best model is provided (second-best models and their weights are discussed in text). A ‘+’ symbol means the predictor was included in the best-fit model, ‘−’ means the predictor was a candidate but not included in the best model, and a shaded box means the predictor was not a candidate for that response variable.

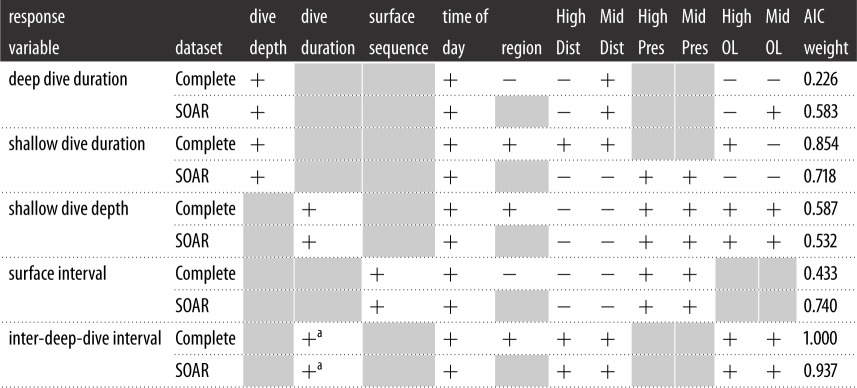

^a^This is the duration of the deep dive preceding the IDDI.

### 3.1. Deep dive duration

**Figure 2.**
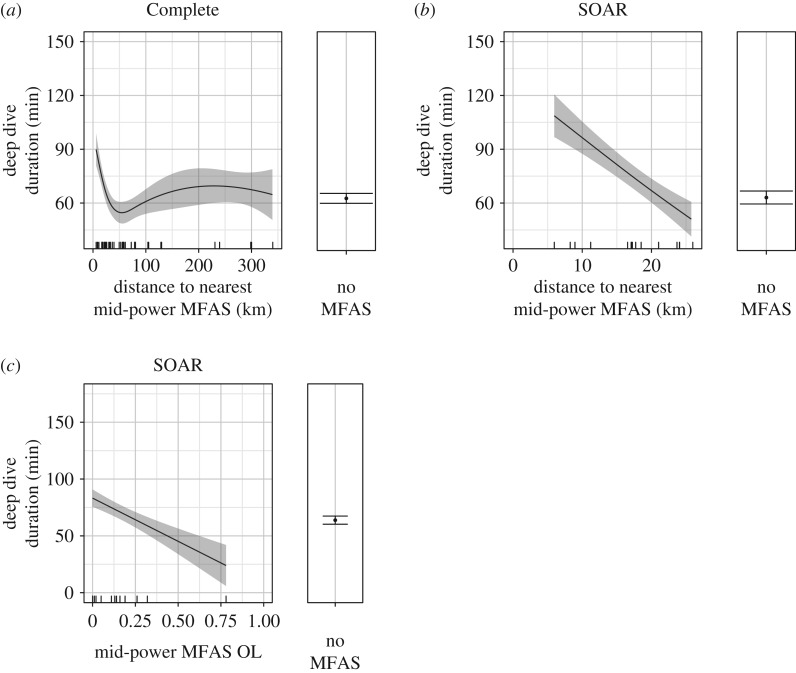
Predictions from the fitted models showing the effect of distance to the nearest mid-power MFAS on deep dive duration using the Complete (*a*) and SOAR (*b*) datasets, and effect of mid-power MFAS OL, or overlap (proportion of the dive during which MFAS was in use), on deep dive duration using SOAR data (*c*), with the predicted dive duration for MFAS-free periods at the right. Solid black lines/dots represent mean predicted values with shaded areas/error bars representing the 95% CI; hash marks along the *X*-axis indicate spread of data. The values of other model predictors were fixed as described in the Material and methods, including the following: dive depth = 1391.5 and mid-power OL = 0.15 (*a*); dive depth = 1519.5 and mid-power OL = 0.11 (*b*); dive depth = 1519.5 and mid-power distance = 17.24 (*c*).

Deep dives became longer as the distance to the nearest mid-power MFAS decreased. Using the Complete dataset, the mean deep dive duration was predicted to increase with proximity to mid-power MFAS from approximately 60 min to approximately 90 min beginning at around 40 km (figure 2*a*). The SOAR dataset predicted that the mean deep dive duration returned to MFAS-free levels by approximately 20 km, after increasing to approximately 107 min with mid-power MFAS at approximately 5 km (figure 2*b*). The second-ranked models added distance to the nearest high-power source, with a comparable AIC weight for the Complete dataset (0.224) but a weight roughly half that of the best model in the SOAR dataset (0.293) (table 4).

There were 10 deep dives exposed to mid-power MFAS and four exposed to high-power MFAS within 20 km, the distance at which both datasets indicated an increase in deep dive duration with the lower power of the two source types. While 10 of these dives were indeed long relative to predicted mean values without MFAS, ranging from a 70.2 min dive with MFAS at 18.5 km to a maximum recorded dive duration of 163.3 min with MFAS at 8.3 km, the remaining four were instead unusually short (39.9, 40.9, 43.0 and 55.6 min). In addition to their brevity, another unusual characteristic of these four deep dives was that they were all shallow relative to the bottom: their maximum depths were more than 500 m above the seafloor at the whale's estimated location, whereas previously published data suggested deep dives are typically to or near the bottom in this region [16].

On SOAR, the best model also predicted that, at a given exposure distance, deep dives become shorter as the amount of overlapping mid-power MFAS (mid-power OL) increased. At the lowest mid-power OL values, the mean deep dive duration was predicted to increase by approximately 30 min over confirmed MFAS-free periods, but to fall below MFAS-free values at mid-power OL values above 0.40 (figure 2*c*).

### 3.2. Shallow dives

**Figure 3.**
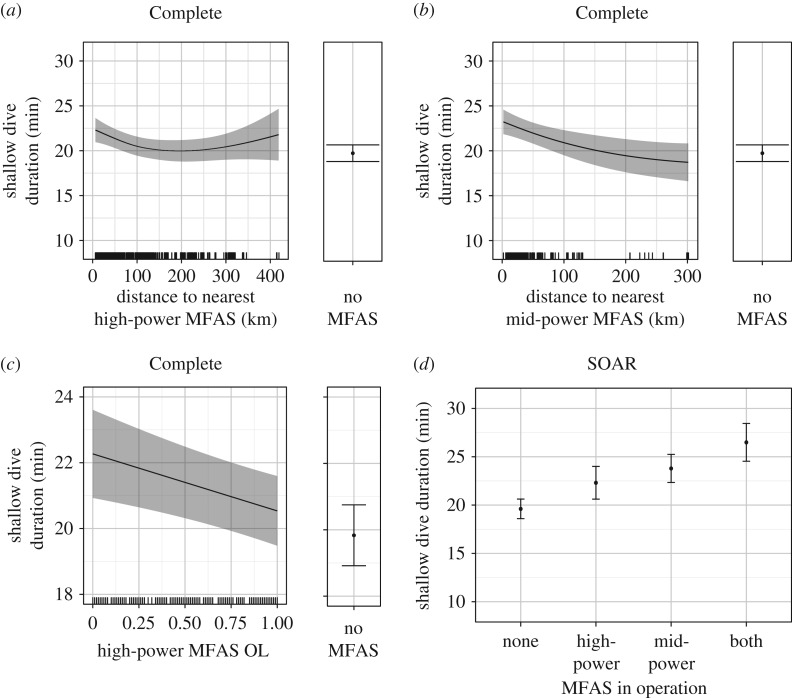
Predictions from the fitted models showing the effect of distance to the nearest high-power (*a*) and mid-power (*b*) MFAS and high-power MFAS OL or overlap (proportion of the dive during which MFAS was in use) (*c*) on shallow dive duration in the Complete dataset, with the predicted duration for periods with no MFAS reported at right, and predictions of the constant effect of MFAS use on SOAR (*d*). Solid black lines/dots represent mean predicted values with shaded areas/error bars representing the 95% CI; hash marks along the *X*-axis indicate the spread of data. The values of other model predictors were fixed as described in the Material and methods, including the following: dive depth = 279.5 and high-power OL = 1 (*a*); dive depth = 279.5 and mid-power OL = 0.28 (*b*); dive depth = 279.5 and high-power distance = 98.01 (*c*); dive depth = 287.5, high-power OL = 1 and mid-power OL = 0.26 (*d*).

Using the Complete dataset, shallow dives were predicted to increase in duration as the distance to both high- and mid-power MFAS sources decreased, beginning at approximately 100 km. Proximity to mid-power MFAS ultimately increased shallow dive duration slightly more than proximity to high-power MFAS at the closest ranges, though the maximum increase was only several minutes in either case (from approx. 20 min without MFAS to approx. 24 min with mid-power MFAS at close range, figure 3*a,b*). At a given distance, shallow dives in the Complete data also increased modestly in duration, to approximately 2 min above MFAS-free levels, as high-power MFAS OL decreased (figure 3*c*). The best model of shallow dive duration received eight times more weight than the second-best model (AIC weight = 0.105), which added mid-power MFAS OL to the best model.

The best model of shallow dive duration on SOAR, where the maximum exposure distances were 51 km and 39 km for high- and mid-power MFAS, respectively, included a presence/absence effect of MFAS use rather than one with dependence on distance. As with the Complete dataset, mid-power MFAS use increased shallow dive duration slightly more than high-power MFAS use (from approx. 19 min in confirmed MFAS-free periods to approx. 24 min with mid-power MFAS), and exposure to both types of MFAS resulted in the longest dives (approx. 26 min) (figure 3*d*). The best model received five times more weight than the second-best model (AIC weight = 0.144), which added high-power MFAS OL to the best model.

**Figure 4.**
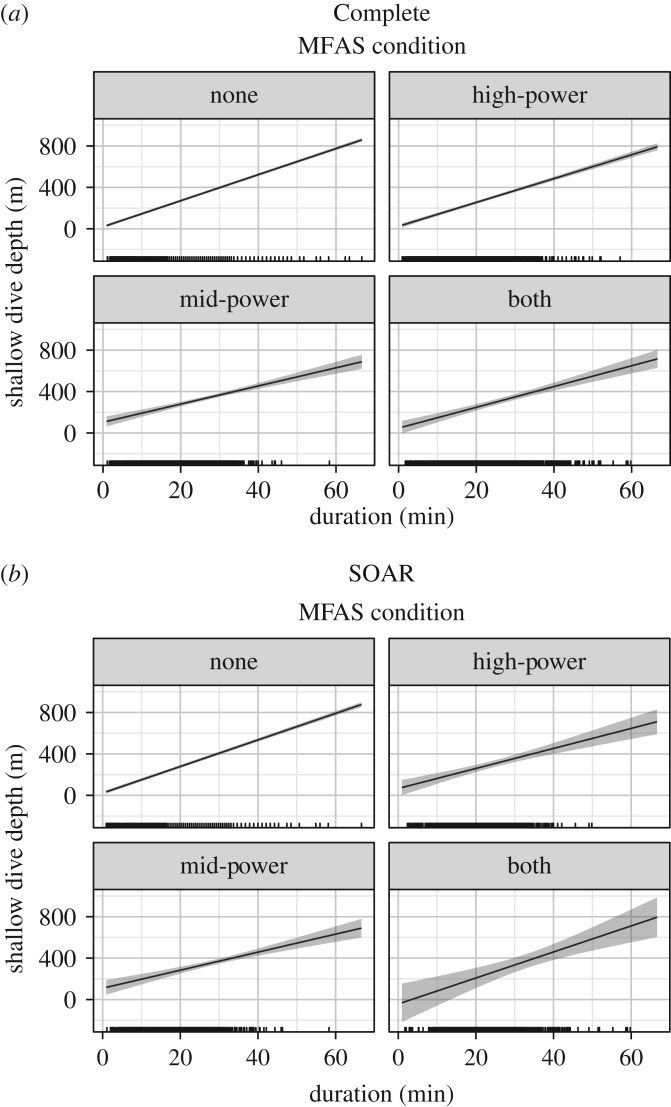
Predictions from the fitted models of shallow dive depth showing the change in the relationship between shallow dive depth and duration as a function of MFAS presence/absence, by type, in the Complete dataset (*a*) and on SOAR (*b*). The 95% CI for the relationship between depth and duration without MFAS (none) is so narrow that it is barely visible. Solid black lines represent mean predicted values with shaded areas representing the 95% CI; hash marks along the *X*-axis indicate the spread of data. The values of other model predictors were fixed as described in the Material and methods, including the following: high-power OL = 1 and mid-power OL = 0.28 (*a*); high-power OL = 1 and mid-power OL = 0.26 (*b*).

Shallow dive depth and duration had a very strong positive linear correlation in all the data, where longer shallow dives were almost invariably deeper. That relationship became more variable when MFAS of either type was in use, and tended to flatten slightly, so that the longer dives seen during MFAS use were often shallower than expected for dives of similar duration when MFAS was not in use, particularly for mid-power MFAS (figure 4*a,b*). This effect was evident in both datasets but most notable on SOAR (figure 4*b*), where both depth and duration of shallow dives were best explained by a presence/absence MFAS effect (figure 3*d*). The full models for shallow dive depth, with MFAS use as a presence/absence effect, received about twice the weight of the second-ranked models (AIC weights: 0.240 Complete, 0.273 SOAR), which both excluded mid-power MFAS OL from the best model.

**Figure 5.**
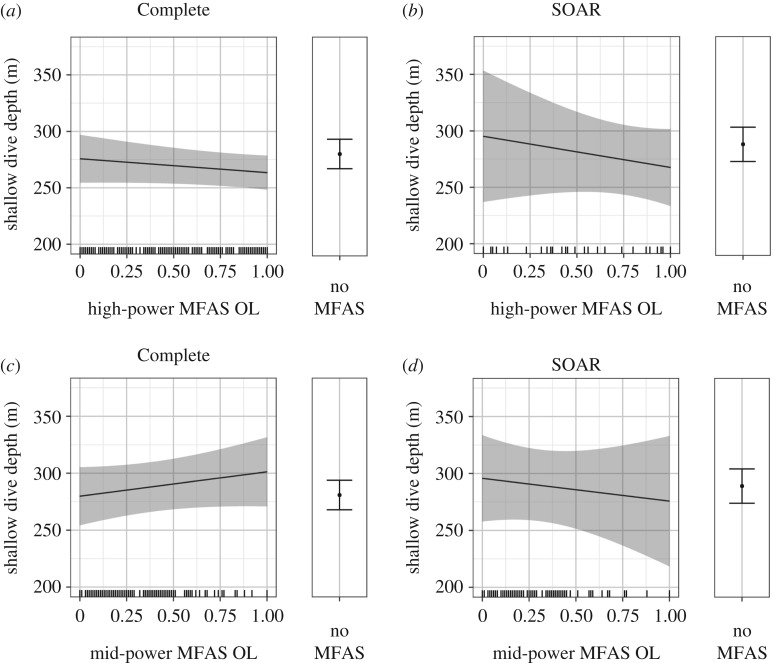
Predictions from the fitted models showing the effect of MFAS OL, or overlap (proportion of the dive during which MFAS was in use), on shallow dive depth as follows: high-power MFAS OL or overlap, in the Complete (*a*) and SOAR (*b*) datasets, mid-power MFAS OL in the Complete (*c*) and SOAR (*d*) datasets. In all plots, solid black lines/dots represent mean predicted values with shaded areas/error bars representing the 95% CI; hash marks along the *X*-axis indicate the spread of data. The values of other model predictors were fixed as described in the Material and methods, including the following: dive duration = 20.78 and high-power presence = 1 (*a*); dive duration = 20.83 and high-power presence = 1 (*b*); dive duration = 20.78 and mid-power presence = 1 (*c*); dive duration = 20.83 and mid-power presence = 1 (*d*).

The effects of MFAS OL on shallow dive depth were generally subtle. In both the Complete (figure 5*a*) and SOAR (figure 5*b*) datasets, shallow dives were slightly shallower and decreased in depth as high-power MFAS OL increased, though values were not far outside those seen without MFAS. Shallow dive depth increased slightly as mid-power MFAS OL increased in the Complete dataset (figure 5*c*), but the opposite relationship was seen on SOAR (figure 5*d*); however, both datasets had wide 95% CIs that largely overlapped with those of MFAS-free periods. This was especially true for mid-power MFAS OL, the one variable that was removed in the second-best models for both datasets.

### 3.3. Surface intervals

**Figure 6.**
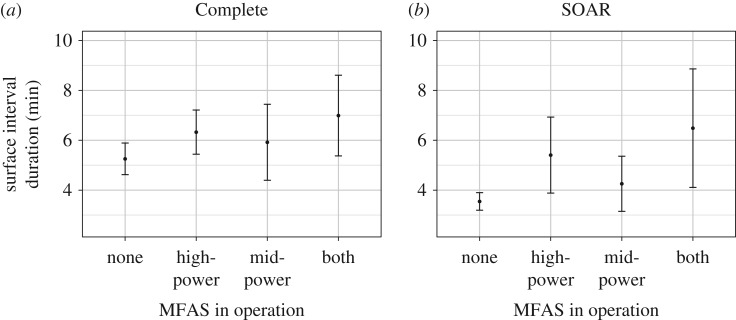
Predictions from the fitted model showing the presence/absence effect of MFAS on surface interval duration in the Complete (*a*) and SOAR (*b*) datasets. Dots represent mean predicted values with error bars representing the 95% CI. For these plots, the values of other model predictors were fixed as described in the methods, including the following: Surface type = ‘Intermediate’.

Surface intervals tended to be longer, but also more variable in duration, during either type of MFAS use (figure 6*a,b*). This effect was most apparent on SOAR, where predicted surface time during confirmed MFAS-free periods was brief and constrained to a very narrow interval (figure 6*b*), relative to both periods with MFAS use on SOAR and periods with no reported MFAS use in the Complete dataset. In both datasets, high-power MFAS use increased the surface interval more than mid-power, particularly on SOAR. The second-ranked models for surface interval, both of which excluded the presence of mid-power MFAS from the best model, received nearly as much AIC weight as the best model in the Complete dataset (AIC weight = 0.428), but much less support than the best model on SOAR (AIC weight = 0.259).

### 3.4. Inter-deep-dive intervals

**Figure 7.**
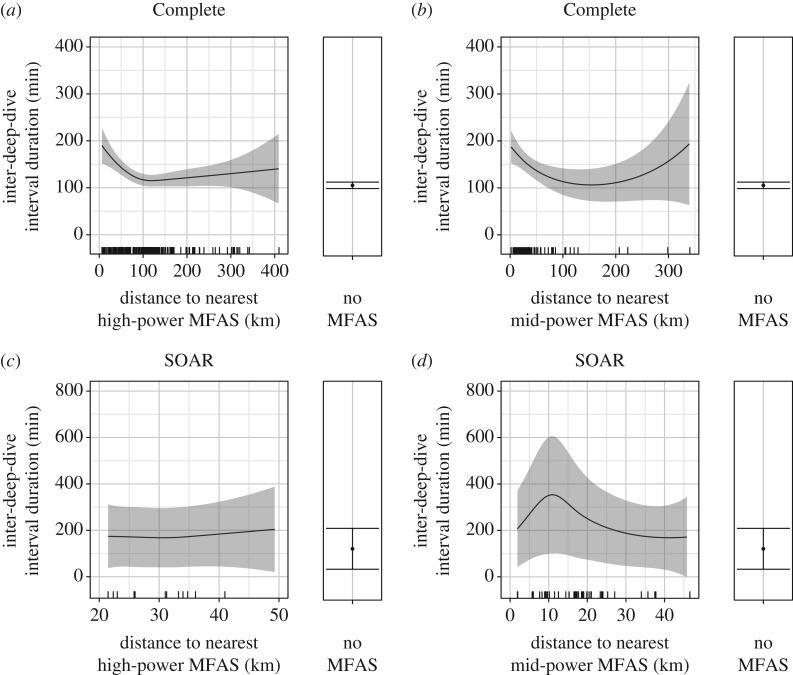
Predictions from the fitted models showing the effect of distance to the nearest high-power (*a*) and mid-power (*b*) MFAS on the IDDI in the Complete dataset, and for high-power (*c*) and mid-power (*d*) using SOAR data, with the predicted IDDI for periods without MFAS at the right. Solid black lines/dots represent mean predicted values with shaded areas/error bars representing the 95% CI; hash marks along the *X*-axis indicate the spread of data. The values of other model predictors were fixed as described in the Material and methods, including the following: previous deep dive duration = 64.45 and high-power OL = 0.39 (*a*); previous deep dive duration = 64.45 and mid-power OL = 0.11 (*b*); previous deep dive duration = 64.51 and high-power OL = 0.46 (*c*); previous deep dive duration = 64.51 and mid-power OL = 0.10 (*d*).

The full models for the IDDI, with MFAS as a distance-mediated effect, were vastly superior to all competing models, with almost all the AIC weight for both datasets. In the Complete dataset, the IDDI became longer with proximity to both types of MFAS beginning at approximately 100 km, reaching approximately 200 min, double the IDDI without MFAS, at the closest distances (figure 7*a,b*). The 95% CIs for the IDDI in the Complete dataset were narrow, both during exposure to MFAS within 100 km, and especially for periods without reported MFAS use.

While exposure distance was also included in the best models of the IDDI on SOAR, the response was much more variable than in the Complete dataset and differed for high- and mid-power MFAS. The mean predicted IDDI during high-power MFAS use on SOAR ranged from 180 to 200 min, approximately 50 min longer than during confirmed MFAS-free periods; however, the distance trend was weak, and the 95% CIs were wide and overlapping for both conditions, spanning approximately 300 min with high-power MFAS use and approximately 200 min without (figure 7*c*). The 95% CI for the IDDI during mid-power MFAS use on SOAR was even wider, reaching a maximum range of nearly 600 min. In contrast to high-power MFAS use, there was a much clearer trend with distance; the mean predicted IDDI during mid-power sonar use on SOAR reached a maximum of approximately 3.5 times the mean IDDI without MFAS with a mid-power source at approximately 10 km (figure 7*d*). Ultimately, the IDDI during high-power MFAS on SOAR may have been better explained as a presence/absence effect that increased the IDDI similarly across the range of exposure distances that occurred on SOAR. However, because our model selection procedure required a single effect type be applied to both MFAS types, the strong distance trend associated with mid-power MFAS use favoured the distance-mediated effect in model selection.

**Figure 8.**
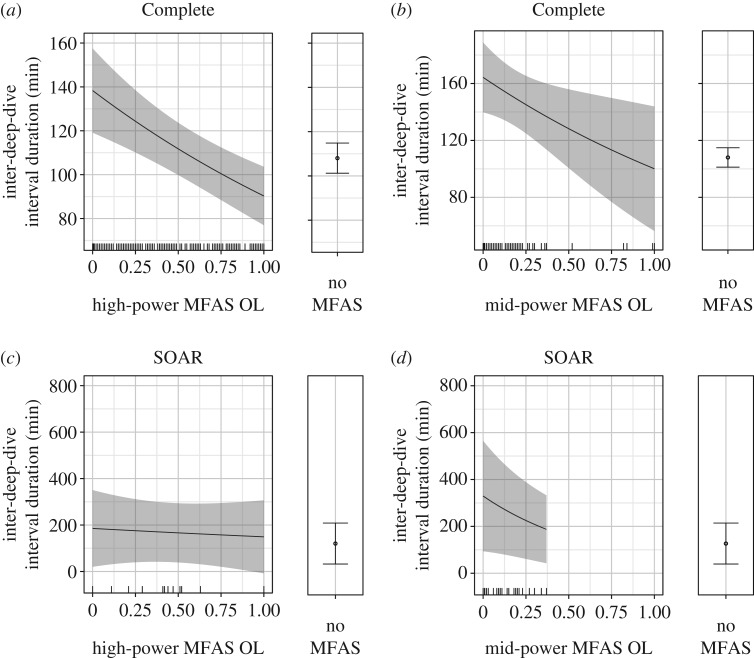
Predictions from the fitted models of the effect of MFAS OL, or overlap (proportion of the interval during which MFAS was in use), on the IDDI: high-power OL (*a*) and mid-power OL (*b*) in the Complete dataset; high-power OL (*c*) and mid-power OL (*d*) on SOAR. Solid black lines/dots represent mean predicted values with shaded areas/error bars representing the 95% CI; hash marks along the *X*-axis indicate the spread of data. The values of other model predictors were fixed as described in the methods, including the following: previous deep dive duration = 64.45 and high-power distance = 98.23 (*a*); previous deep dive duration = 64.45 and mid-power distance = 27.29 (*b*); previous deep dive duration = 64.51 and high-power distance = 31.14 (*c*); previous deep dive duration = 64.51 and mid-power distance = 17.20 (*d*).

The IDDI decreased as OL with both types of MFAS increased in the Complete dataset. The mean predicted IDDI ranged from approximately 140 min at high-power OL values near zero to approximately 90 min as high-power OL approached one (figure 8*a*). Mid-power MFAS OL decreased the IDDI from approximately 160 min near zero to approximately 100 min as mid-power OL approached one. However, estimates for the IDDI at mid-power OL above 0.3 had extremely wide confidence intervals, given sparse data (figure 8*b*). On SOAR, the IDDI also tended to decrease as MFAS OL increased, with values ranging from approximately 190 min to approximately 150 min as high-power OL increased from zero to one (figure 8*c*), and from approximately 310 min to approximately 200 min as mid-power OL increased from zero to 0.3, beyond which there were no data to inform predictions (figure 8*d*). MFAS-free values on SOAR centred around approximately 120 min, though all predictions on SOAR had wide and often overlapping confidence intervals, particularly during MFAS use where samples were small.

The overall mean deep dive cycle (IDDI + preceding deep dive duration) for all whales when no MFAS was reported was 175 min (range 33–680 min, *n* = 1647), corresponding to approximately 8 deep dives per day. In the full BL data, there were 37 (1.8% of total) IDDIs longer than 300 min, approximately three times the mean predicted IDDI without MFAS (figure 6). These IDDIs, in the range of 301–641 min, corresponded to an estimated loss of three to six deep dives, assuming a typical deep dive cycle of 175 min. Seventy per cent (26 of 37) of these very long IDDIs included MFAS of one or both types. There were eight whales that each had more than 100 deep dive cycles in the Complete data. These whales averaged 6% of dive cycles with high-power MFAS within 100 km (range 4–14% per individual), and 4% of dive cycles with mid-power within 100 km (range 1–7% per individual). Dive cycles with MFAS within 20 km, the distance where the IDDI generally increased 2–3 times, though occasionally by a factor of six, were infrequent. They accounted for a maximum of 2% per individual with high-power MFAS, and 3% with mid-power MFAS, for these eight whales.

## 4. Discussion

This study analysed a large collection of dive and surfacing behaviour data from Cuvier's beaked whales with and without exposure to two common military sonar systems: high-power, hull-mounted, surface-ship MFAS and mid-power, helicopter-deployed dipping MFAS. In addition to the time of day, geographical area and several within-individual behavioural correlates, MFAS variables were included in the best models for every behaviour type tested, both on and off SOAR. Distance-mediated effects that increased with MFAS proximity were evident for some behaviours during MFAS use up to 100 km away. While high-power MFAS was often associated with effects over greater distances than mid-power MFAS, responses to mid-power MFAS were often more pronounced at comparable closer ranges, and presumably at lower received levels. This also suggests that an animal exposed at a given received level from a close mid-power source may react more strongly than it does when exposed at the same received level from a more distant high-power source. Deep dives, shallow dives and surface intervals all tended to become longer as distance to MFAS decreased. In combination, these effects contributed to longer intervals between the deep dives that are typically associated with foraging in this species, and thus foraging disruption was probable during and following MFAS use.

Several previous studies have suggested that beaked whales increase the duration of deep dives in response to high-power, hull-mounted MFAS signal types [10–12]. Distance to high-power MFAS was not included in the best models for deep dive duration, though there were few close-range high-power exposures in these data: only 11 of 214 deep dives exposed to high-power MFAS were within 25 km of the source, and none of these were within 15 km. The previous record for mammalian dive duration (137 min) was from another Cuvier's beaked whale tagged as part of this work in 2010 [16]. Comprehensive MFAS use data were not available from 2010, so tags deployed that year were excluded from this analysis; however, a review of the archives around the time when the 137 min dive occurred revealed 45 min of high-power sonar use in the middle of the dive, approximately 9 km from the whale's predicted location. Thus, very close-range exposures to high-power MFAS during deep dives may increase their duration, but the current data were insufficient to detect a trend.

One possibility is that there were so few high-power MFAS exposures at close ranges because these whales, many of which exhibit high site fidelity to this regularly used MFAS training area [16,19], avoid diving deeply, or perhaps begin to displace, when military surface ships are near. Testing this hypothesis is important because it suggests that not only the use of MFAS, but also other Navy activities that precede or succeed its use may influence the behaviour of these whales. This hypothesis could be evaluated using tag data and ship tracks to compare deep dive and movement rates when military ships were within 15 km, but not using their MFAS, to behaviour when there were no military ships nearby.

In contrast, proximity to mid-power MFAS was associated with an increase in deep dive duration in these data, where 16 of 45 deep dives that coincided with mid-power MFAS use were within 25 km of the source, with a minimum estimated distance of 6 km. The longest deep dive in this study, lasting 163 min, occurred while the whale was intermittently exposed to mid-power MFAS at distances of 8–12 km. While it may be possible for a whale to avoid conducting a deep dive while a surface ship is nearby, the same is not true for helicopters, which acoustically may appear without warning. Surface ships typically broadcast MFAS for extended periods while moving; thus whales probably know roughly where the ship is when exposure begins and how the ship's position is changing through time, and can use this knowledge to mediate their response. Helicopters deploy MFAS from a hover in bouts generally lasting under 20 min, moving rapidly between sequential deployments in an unpredictable pattern, and thus whales may react more strongly to these sudden, close-range exposures even though their duration of use and source level (217 dB) are generally well below those of a ship's MFAS (235 dB) [27]. The difference in these responses underscores the importance of how the source is used, in addition to sound levels (source or received) and distance, in predicting whale behaviour, particularly for whales that are probably familiar with both MFAS types.

One mechanism of sonar-induced behavioural response that has been proposed to explain the coincidence of strandings and sonar has been rapid ascent from a deep dive, resulting in the deleterious effects of gas bubble formation especially when tissues are supersaturated with nitrogen [5,7]. The dive data from this study do not include ascent rates, so we cannot directly address this hypothesis, other than to observe that none of the tagged whales displayed any post-MFAS behaviour that was indicative of morbidity. However, one might hypothesize that if the typical response to MFAS was to abort a dive and surface abruptly, then when MFAS exposure is frequent, dive durations would tend to be shorter than average, which we did not observe. While unusually short deep dives did occasionally occur during MFAS use, they were much less common than extended deep dives. These short deep dives were also unusual in that they were shallow relative to local bottom depth. This suggests these dives never reached primary foraging habitat for whales in this region, where deep dives typically approached the seafloor [16], rather than that they were aborted after reaching typical foraging depth. Assuming whales may alter their behaviour during MFAS in part to reduce the intensity of the sound they are exposed to and that sound levels are likely to be higher near the surface, particularly at close range, remaining at depth as long as possible when already near the bottom, remaining mid-water if exposed on deep dive descent or conducting unusually deep non-foraging dives may all help reduce sound exposure, and may explain why both deep and shallow dives typically increased in length with MFAS proximity.

Remaining at depth during MFAS exposure may reduce received levels, but that response could increase the risk of DCS symptoms, especially if the whales were at depths above the presumed depth of alveolar collapse, in the region where nitrogen continues to be transferred from the lungs to the tissues [13,33]. Spending additional time below the depth of lung collapse can also exacerbate DCS risk by increasing the transfer of nitrogen from tissues with fast uptake rates (e.g. muscle) to those with slow uptake rates (e.g. fat), which will lead to greater levels of nitrogen accumulation [13]. These negative impacts of increase in the time at depth may therefore limit the extent whales use depth to mitigate sonar exposure. In the absence of MFAS, shallow dive duration and depth were related with almost no variability, i.e. unusually long shallow dives were also unusually deep. But as shallow dives became longer during MFAS use, they did not become commensurately deeper, and were still much shorter and shallower than typical deep dives without MFAS. This pattern, coupled with the tendency for the IDDI to increase during MFAS use, suggests that it is unlikely that whales exposed to MFAS during a shallow dive routinely ‘switched’ to a deep dive (i.e. descended deep enough for long enough for the dive to have been statistically classified as a deep dive for that individual). Schorr *et al.* [16] noted significant differences in the duration of surface intervals based on when they occurred in the deep dive cycle: the final surfacing just prior to each deep dive was significantly longer than surface intervals that separate shallow dives. This effect, also apparent in the inclusion of the Surface Sequence predictor in the best model of surface interval duration here (table 4), suggests that whales need this extra 1–2 min of surface time to prepare for deep dives, and that they cannot necessarily shift from shallow to deep diving once a dive has commenced. It is also possible that whales use longer, but not deeper, shallow dives during MFAS use to displace horizontally, rather than vertically, to mitigate sound exposure. This may be supported by the finding that shallow dives that occurred during prolonged high-power MFAS use (with high-power OL at or near 1) tended to be shallower than shallow dives without MFAS (figure 4*c,d*). The use of modelled location data for these tags, with a minimum possible time step of 30 min, and coarse MFAS positions, precluded the inclusion of displacement metrics in the behavioural models used here. However, an independent analysis of displacement as a function of MFAS use could provide some insight into the role horizontal displacement plays in response, particularly if paired with the higher-resolution MFAS movement data that are available for many operations on SOAR. While this is possible using the modelled spatial data, improved satellite tag technology with high-resolution, GPS-derived movements would provide a more robust means of studying displacement during exposure, and potentially allow for both vertical and horizontal movements to be modelled simultaneously.

The demands of increased dive durations may drive the increase in surface intervals also seen during MFAS use. A surface interval effect was most evident on SOAR, where the night-time mean surface time more than doubled from approximately 3 to over 6 min when both types of MFAS were active, though increases during only one type were more modest, at roughly 2 min for high-power and 1 min for mid-power MFAS. During MFAS use, and even following the most extreme deep dives, whales appeared to conduct more of these modestly longer surface intervals, contributing to extended IDDIs, rather than to dramatically extend a single surface interval. While long surface intervals were uncommon in all the data, they were occasionally seen, particularly at night when MFAS was not in use. Also, back-to-back deep dives, which were typically separated by a single very long surface interval (median duration = 46.4 min, range 1.3–115.7 min), were observed on 56 occasions without MFAS, but this pattern was observed only once when MFAS of either type was active within 100 km, with the intervening surface interval lasting just over 5 min. This may suggest that whales increase surface time only as much as is necessary while MFAS is in use, a response that may mitigate sound exposure at closer ranges. Avoiding prolonged surface intervals, even when they might be physiologically advantageous (e.g. to support the gas exchange demands of prolonged dives, with or without increased displacement) also lends support to the risk-disturbance hypothesis, which suggests whales may respond to MFAS in a manner that is shaped by a species-specific anti-predator strategy, which for beaked whales probably involves escape through deep and prolonged diving [34].

Another noteworthy prediction of the surface interval models was that these whales appeared to conduct much shorter surface intervals during confirmed MFAS-free periods on SOAR than during periods without reported MFAS use in the Complete dataset. This stark difference in presumed MFAS-free surface behaviour was surprising, because geographical area was not selected as a predictor of surface interval in the best model of the Complete dataset, suggesting the behaviour change was associated with the range itself, and not the basin in which SOAR is located. While there may be other naturally occurring factors not accounted for in this model that affect surface interval, one possibility is that whales routinely limit their surface time on SOAR to avoid increased vessel traffic or other noise-generating, surface-based operations that also occur there regularly [21]. These include explosions and small arms fire, both of which could potentially be detected in the hydrophone archive, and thus their effect on whale behaviour could potentially be assessed using this method.

The tendency for deep and shallow dives in some models, and the IDDI in all models, to become shorter as the amount of overlapping MFAS (OL) increased is at least partly explained by the fact that these behaviours tend to become longer during exposure, but the durations of MFAS bouts are fairly consistent and independent of the whales' behaviours; thus the relationship between these factors tends to be inverse. Inaccurate reporting in SPORTS, which included eight MFAS bouts that were improbably long (800–1440 min) but for which the actual duration could not be verified, could also have created behaviours with high OL values that occurred when MFAS was no longer active, artificially reducing the apparent effect of high OL values in the Complete dataset. However, these inaccurate records represented a small proportion of the total data, and MFAS OL was also included in several of the best models on SOAR, where there were no unusually long, unverified bouts of use, reducing the likelihood that inaccurate reporting was a primary driver of these results. It is possible that whales alter their behaviour most strongly when MFAS use first commences or is being used intermittently, increasing the likelihood of a startle response [35]; both are cases where OL is less than 1, contributing to this effect.

While the same inherent inverse relationship between behavioural event duration and MFAS bout duration also contributed to an association between unusually short behaviours and high MFAS OL values, there is probably also a real underlying response pattern reflected in this relationship. For example, a review of 10 unusually short deep dives that occurred during mid-power MFAS use on SOAR revealed that in seven of them, ranging in distance from 11 to 30 km to the source, the whale exhibited the aforementioned unusual pattern of descending to lower mid-water (ranging from 63 to 79% of local bottom depth) rather than to typical foraging habitat near the bottom. In all of these cases, the dive either commenced just after MFAS onset, and thus may have been a vertical avoidance response rather than a true foraging attempt, or MFAS commenced shortly after the whale left the surface, and the whale may have aborted a would-be foraging attempt before reaching the bottom; in all cases, MFAS OL values were at or near one, with MFAS starting early and continuing intermittently through all or most of the ensuing truncated deep dive. Six of these 10 unusually short deep dives during MFAS were conducted by the same whale, suggesting some individuals may favour a response that is not typical of the larger population, and highlighting the importance of considering individual variation when characterizing responses.

The other behaviour that became unusually short at high OL values was the IDDI during high-power MFAS use in the Complete dataset. A review of 13 unusually short IDDIs (less than 90 min) with MFAS OL above 0.8 revealed two interesting patterns. Five of these short IDDIs ended when the whale conducted an unusually short, mid-water deep dive with MFAS still in use. As noted above, these dives probably represent a response to MFAS, and not a true foraging effort; if so, IDDIs separated by these aberrant deep dives should actually be summed into a single extended IDDI, at least where the IDDI is being considered a proxy for foraging disruption. Three other of these short IDDIs followed unusually long IDDIs that also overlapped with MFAS. These cases may represent an increase in the foraging rate following the initial, more typical, response (i.e. delayed foraging), despite the fact that MFAS was still in use. Because the MFAS data used in this analysis provided only the position of the source when transmission began, ships could move considerable distances during prolonged exercises, and if the ship moves away or does not come closer, whales may well resume foraging at, or even above, MFAS-free rates while the source is still active, creating unusually short IDDIs with high MFAS OL. These examples provide two potential mechanisms by which extended use of MFAS could be associated with an apparent reduction in the IDDI, as these models suggest.

Response patterns in deep dives and the IDDI associated with MFAS OL help interpret some of the high variability seen in the predictions on SOAR, particularly for the IDDI, where sample sizes were much smaller. They also underscore the value of coupling passive acoustic vocal data with dive records from these tags in the future to determine whether a given deep dive was actually associated with evidence of foraging activity near the whale's location. M3R detection archives have already been used to derive a risk function for MFAS-associated foraging disruption for Blainville's beaked whales at AUTEC [17]; they could also be used to identify deep dives in this dataset that occurred without echolocation clicks on nearby hydrophones to refine the approach here. The use of extended-duration tags with acoustic recording capability, currently under development, could provide vocalization data from the tagged whale itself, and would be the best way to verify foraging activity during deep dives in the future.

## 5. Conclusion

Exposure to both high- and mid-power military MFAS systems was associated with a variety of behavioural changes in satellite-tagged Cuvier's beaked whales in the Southern California Bight. Responses that increased with proximity were evident up to 100 km away in this large dataset that included sizeable samples of behaviour from 16 whales during periods both with and without MFAS use, despite the relative coarseness of both the behavioural and MFAS data used in this analysis. Responses were often more pronounced for mid-power rather than high-power MFAS use at comparable close distances even though received levels, the metric by which effects are currently estimated for permitting purposes [21,36], were probably considerably lower for mid-power MFAS. With other behavioural predictors taken into account (including time of day, region and individual behavioural patterns), deep dives, shallow dives and surface bouts all tended to increase in duration during MFAS use. Variability in predictions was probably associated with how the source was used (continuous, more predictable bouts associated with ships versus intermittent, less predictable bouts associated with helicopters), and the relative timing and duration of MFAS use (onset prior to, early or late in the behaviour; initial or prolonged use), and in some cases may reflect individual behavioural preferences. A detailed review of specific responses within these data that do not follow predicted patterns, particularly on SOAR, where detailed MFAS source geometry can be recreated and ping-by-ping sound fields modelled, may provide additional insight into circumstances that increase or reduce responsiveness.

Exposure to MFAS within 100 km at any point in the deep dive cycle was usually associated with an increased IDDI (and therefore a reduced deep dive rate), a proxy for foraging disruption in this species. In at least some specific cases where the IDDI was instead reduced during MFAS use, there is evidence that the whale did not actually resume foraging on its next deep dive, and thus the foraging disruption effect may be even stronger than these results suggest. Increased IDDI is the response most likely to have long-term fitness consequences if exposures happen frequently, though these data suggest that exposures associated with the greatest increases in the IDDI are relatively uncommon. The relative strength of the increased IDDI response (i.e. that models of the IDDI including MFAS variables were so superior to models without them), which could also potentially be documented through changes in vocal activity in the archive [9], supports the continued use of foraging disruption in Population Consequences of Disturbance models being developed for this and other beaked whale populations on sonar training ranges [17,18]. However, given that 30% of extremely long IDDIs did not coincide with reported high- or mid-power MFAS use (three of which were confirmed free of MFAS), there are likely to be other factors not included in this analysis that can significantly extend the deep dive cycle. An attempt should be made to identify if any of these long cycles were potentially associated with other anthropogenic activities through a review of operational schedules and the archive. Benthic prey data, which are difficult to collect at the depths these whales typically forage, would also greatly improve our understanding of the deep dive cycle in this species. Given these whales' preferential use of this regularly disturbed area, it does beg the question of whether there are local ecological advantages that help to offset the presumed cost of inhabiting a busy military training range.

The initial impetus to study the behaviour of beaked whales exposed to MFAS has been to understand the mechanism underlying sonar-associated stranding events, in hopes of mitigating against them in the future. There have been no documented sonar-associated mass stranding events in southern California [8], and while this does not necessarily mean they have never occurred, particularly given the long history of sonar use and the low odds of detecting a stranding in this offshore area [37], none of the whales tagged in this study stranded or closely approached land despite some exposures estimated at a distance of just a few kilometres. While some responses exceeded the behavioural extremes previously established for this species [14,16], the whales went on to resume apparently normal diving patterns until the next time they were exposed. Thus, the primary impacts of MFAS use on this sonar-savvy population are likely to be cumulative, rather than acute, in nature. Ultimately, these data did not provide conclusive evidence of how MFAS causes whales to strand, but rather many examples of sonar-associated behavioural changes that did not, potentially helping to narrow the focus of future studies. While this study has yet to unmask the causal mechanism behind MFAS-related stranding events, it does suggest that responses are predicated on more than just received levels, and that an animal's familiarity with these sources may mediate its responsiveness.

## Supplementary Material

Extended dive trace with intermittent sonar exposures

## Supplementary Material

Spectrogram of generic MFAS signal

## Data accessibility

The data and the code required to analyse them have been deposited in the Dryad Digital Repository. The data are stored as comma-delimited text, with a separate file for each response variable and dataset. Each row of data in each file represents a behavioural event with the MFAS use summarized as described in the Material and methods. The code has been compiled in a pdf file. These materials, and a document describing the contents of each data field, are available at: http://dx.doi.org/10.5061/dryad.125g5 [31].
