# Supplementary figures and images for: Diving behaviour of Cuvier's beaked whales exposed to two types of military sonar
Source: R Soc Open Sci. 2017 Aug 30;4(8):170629. doi: 10.1098/rsos.170629 (PMC5579120; doi:10.1098/rsos.170629)

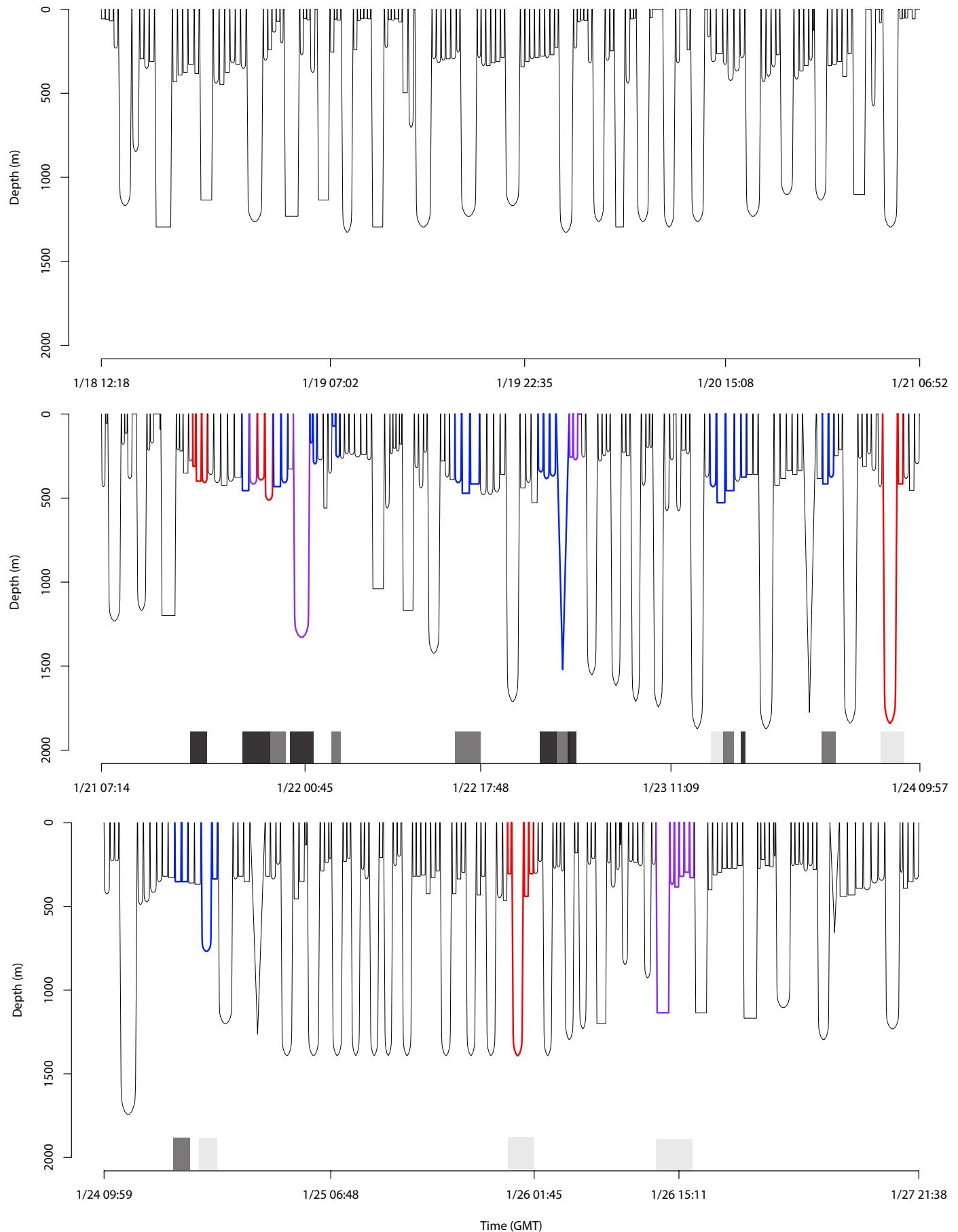

Supplement: Extended dive trace with intermittent sonar exposures [file rsos170629supp1.pdf]

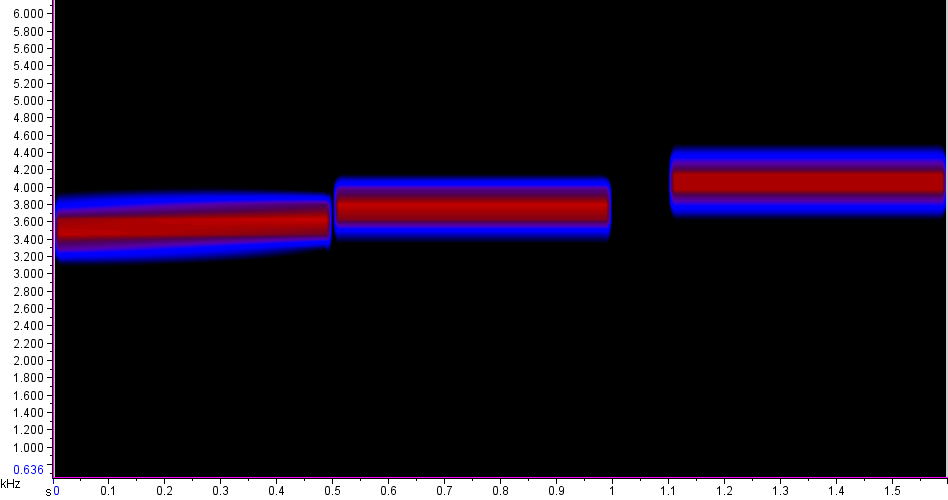

Supplement: Spectrogram of generic MFAS signal [file rsos170629supp2.png]
